# Giant prostatic hyperplasia in a 54-years old patient treated by prostate artery embolization: case report

**DOI:** 10.3389/fruro.2024.1446650

**Published:** 2024-11-29

**Authors:** Nicolas Villard, Georgia Tsoumakidou, Paul C. Moldovan, Rémy Rosset, Olivier Rouvière, Hakim Fassi-Fehri, Gaële Pagnoux

**Affiliations:** ^1^ Department of Medical Radiology, Service of Radiology and Interventional Radiology, Lausanne University Hospital and University of Lausanne, Lausanne, Switzerland; ^2^ Hospices Civils de Lyon, Department of Medical Radiology, Service of Radiology and Interventional Radiology, Hôpital Edouard Herriot, Lyon, France; ^3^ Hospices Civils de Lyon, Service of Urology and Transplantation, Hôpital Edouard Herriot, Lyon, France

**Keywords:** prostatic hyperplasia, lower urinary tract symptoms, interventional radiology, endovascular procedures, therapeutic embolization, transurethral resection of prostate

## Abstract

Giant prostatic hyperplasia (GPH) is defined as benign prostate hyperplasia (BPH) of more than 200 ml. It is a challenging condition because transurethral resection is classically indicated for prostate volume less than 80 ml and open adenectomy remains the gold standard therapy for GPH. Herein, we present the case of a 54-years old male with giant prostatic hyperplasia (total prostate volume of 265 ml) causing lower urinary tract symptoms (LUTS) and recurrent episodes of acute urinary retention. The patient refused the surgical adenomectomy and underwent bilateral prostate arteries embolization (PAE). Post embolization period was uneventful. Total prostate volume decreased progressively and LUTS disappeared. At more than 5 years follow-up the patient remains still asymptomatic, despite the discrete regrowth of the prostate detected on imaging. This case report suggests that PEA may be a good alternative to open surgery for patients with symptomatic GPH.

## Background

Benign prostatic hyperplasia (BPH), causing lower urinary tract symptoms (LUTS), is a major health concern with increasing prevalence due to ageing of the population. It affects the Quality of Life (QoL) in 15% to 60% of men (1). Both medical and surgical therapies for symptomatic BPH are effective, but they are associated with significant morbidity rates and some degree of sexual dysfunction (2).

For giant prostatic hyperplasia (GPH; >200 ml), a rare subtype of BPH, surgical options are limited and open prostatectomy still remains the recommended treatment but harbors a higher complication rate (3). Prostate arteries embolization is a minimally-invasive procedure alternative to surgery, especially for sexually active adults suffering of LUTS, with no maximum prostate volume threshold (4).

We present a case report that highlights the prostate embolization as treatment of GPH.

## Case presentation

### Initial presentation

We share the case of a 54-year-old male known for BPH and LUTS. Recently, he had several episodes of acute urinary retentions, with the need of permanent indwelling urinary catheter, despite well followed medical therapies (Silodosine, per os, 8 mg once a day; Pygeum africanum, per os, 50 mg twice a day).

### Diagnostic assessment

**Figure 1:**
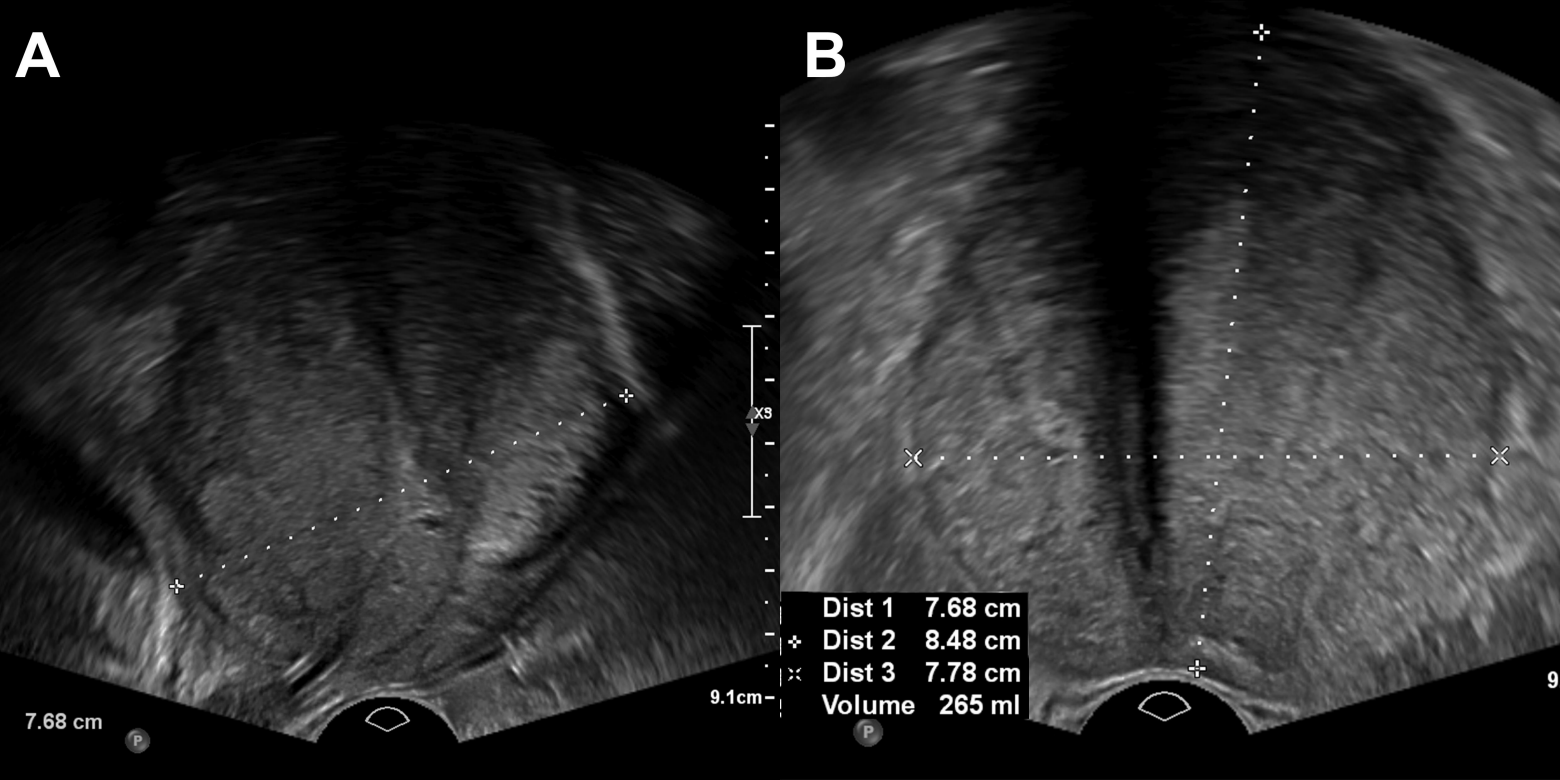
Transrectal ultrasound with sagittal **(A)** and axial **(B)** planes showing a volume of 265 mL.

Prostate volume assessed by TRUS was 265 ml (**Figure 1**). Uroflowmetry was not possible before embolization because of the presence of an indwelling urinary catheter. The prostate-specific antigen (PSA) level was 10.1 ng/ml (PSA density of 0.039 ng/ml^2^), and the prostate was not suspicious at digital rectal examination. Multiparameter prostate magnetic resonance imaging (MRI) showed no suspicious lesion and confirmed prostate volume. The patient had history of negative prostate biopsy 7 years ago. QoL score was 6. International Prostate Symptom Score (IPSS) was not calculated because of the urinary indwelling catheter. He refused surgical adenomectomy.

Decision to perform PAE was taken in a multi-disciplinary basis. Patient informed consent was obtained before treatment. A contrast-enhanced high resolution angio-CT (after sublingual administration of alcoholic solution of trinitrine 0.30 mg) was obtained to evaluate the arterial prostatic anatomy. Renal function was normal (GFR of 59 ml/min). Routine urine culture performed 10 days before PAE revealed asymptomatic infection (Staphylococcus epidermidis 10^7^ CFU/ml and Acinetobacter baumanii 10^7^ CFU/ml), and a treatment of oral cotrimoxazole was started 2 days before PAE for a total of 10 days.

### Treatment

**Figure 2:**
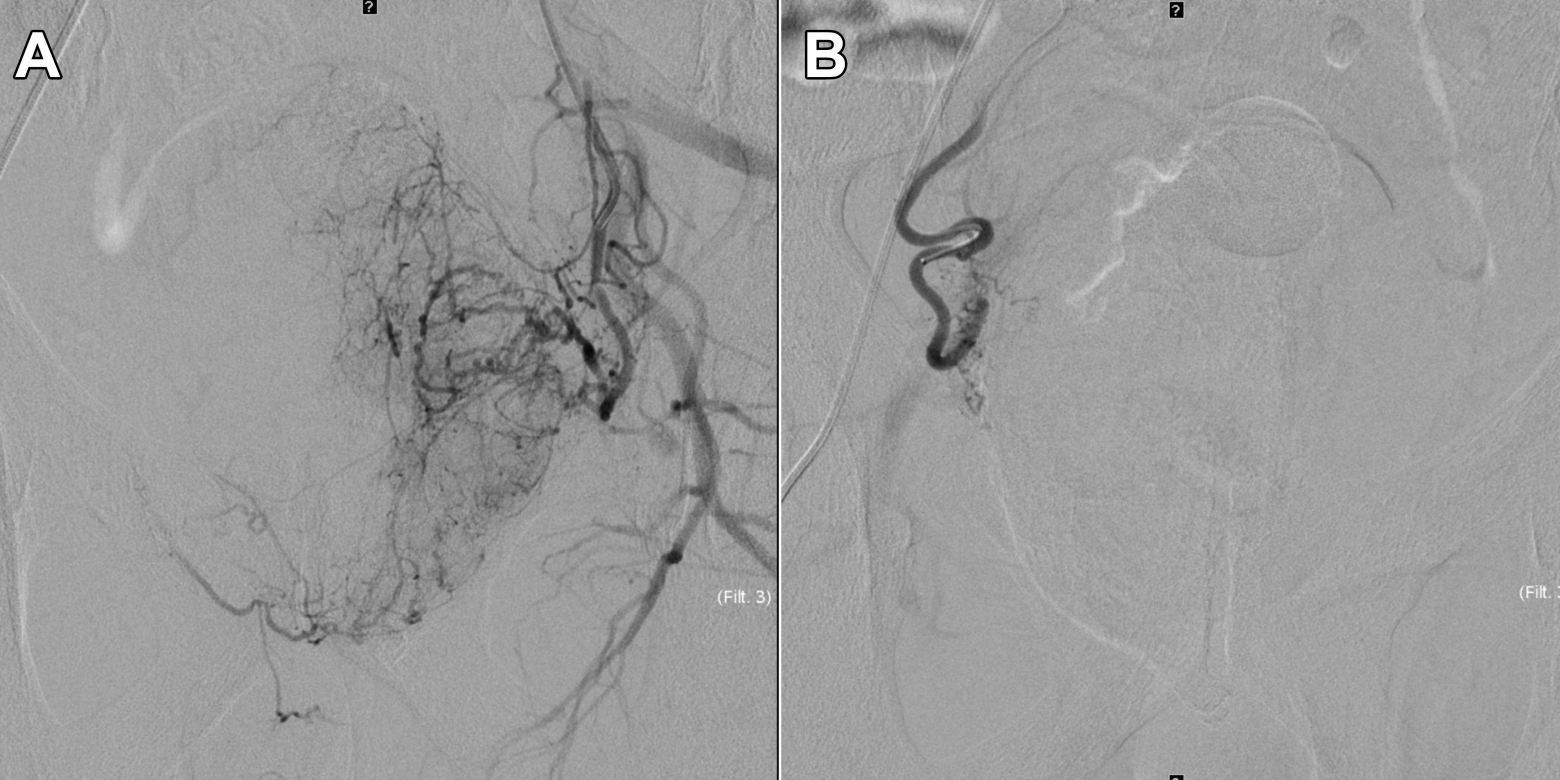
Digital subtraction angiogram (DSA) with the microcatheter positioned in the right **(A)** and then in the left **(B)** prostatic artery, just before injection of microspheres.

The patient was hospitalized the day of the intervention. The procedure took place in an interventional radiology suite, with conscious sedation and local anesthesia. A 5F arterial access (right common femoral artery) was performed. Under fluoroscopic guidance, both prostatic arteries were microcatheterized (**Figure 2**). The microcatheter was advanced as distal as possible selectively in each prostatic artery. After confirming the absence of non-target perfusion by angiogram and conebeam-CT, each half of the prostate was embolized using 300-500 μm spheres (Embospheres, Merit Medical Systems, South Jordan, UT). Arterial access was finally closed using a mechanical sealing device (Angioseal, Terumo Medical, Somerset, NJ). The patient was asked to stay in a supine position for the next 4 hours and then was discharged. He only reported a feeling of pelvic heaviness without any pain that disappeared a few days later. There was no macrohematuria nor blood in the stool.

### Follow-up

**Figure 3:**
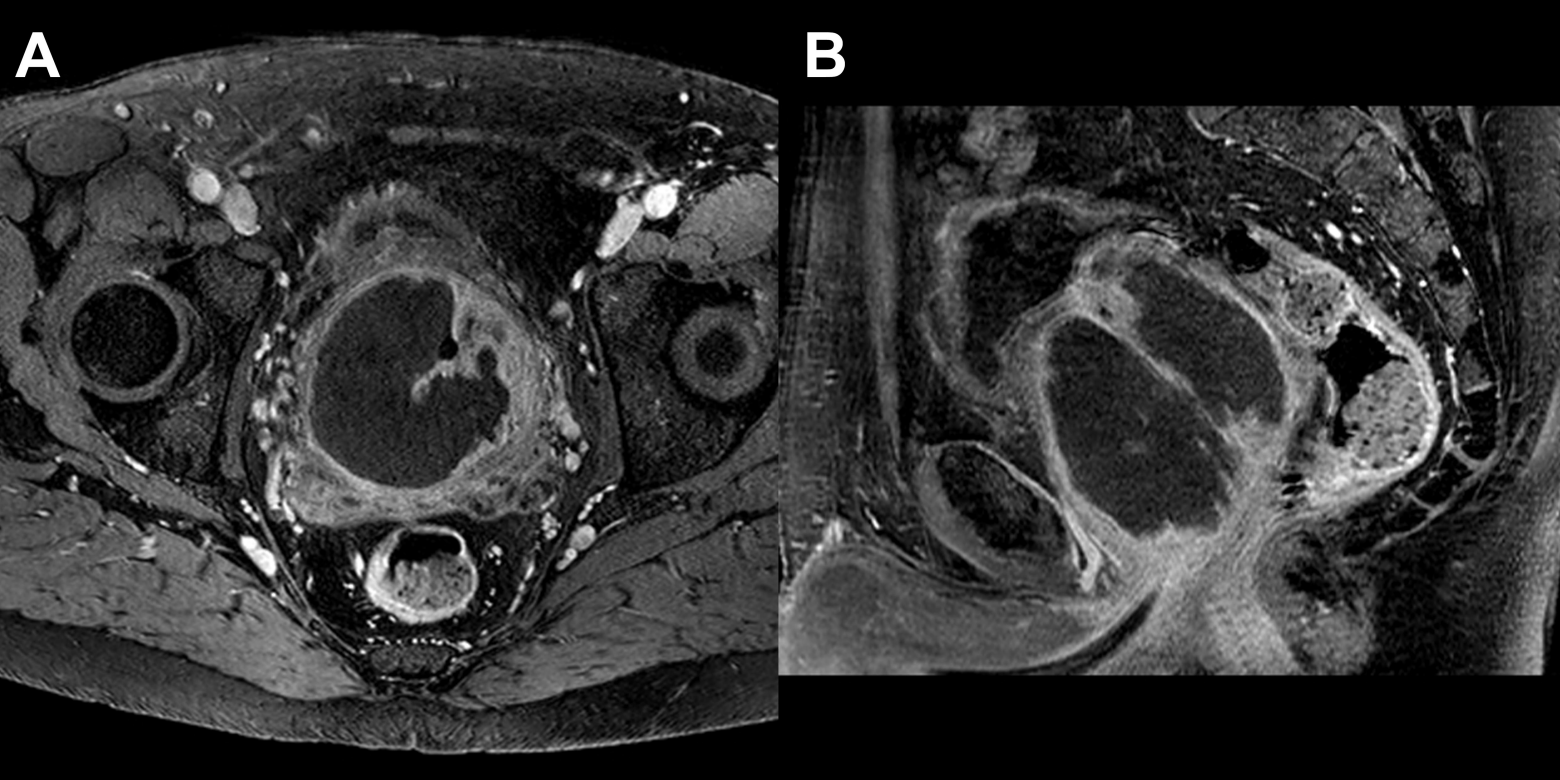
MRI 4-days after PAE: T1 FS after injection of contrast medium shows subtotal devascularization of the transitional zone of the prostate on axial **(A)** and sagittal **(B)** planes.

A prostate MRI, performed 4 days after PAE, showed large necrosis of the transitional zone, with a right-side predominance (**Figure 3**). Post embolization period was uneventful. Urinary indwelling catheter was successfully removed at day 12. The medication for LUTS was discontinued after 1 month. An uroflowmetry was performed just after catheter removal, with peak flow rate (Qmax) at 10.8 ml/s, and no post-void residual volume. After 6 weeks, transrectal ultrasound (TRUS) revealed a volume of 182 ml (- 31%) and a smaller protrusion of the prostate at the level of the bladder neck. Qmax improved to 24.8 ml/s. PSA dropped to 3.86 ng/ml.

**Figure 4:**
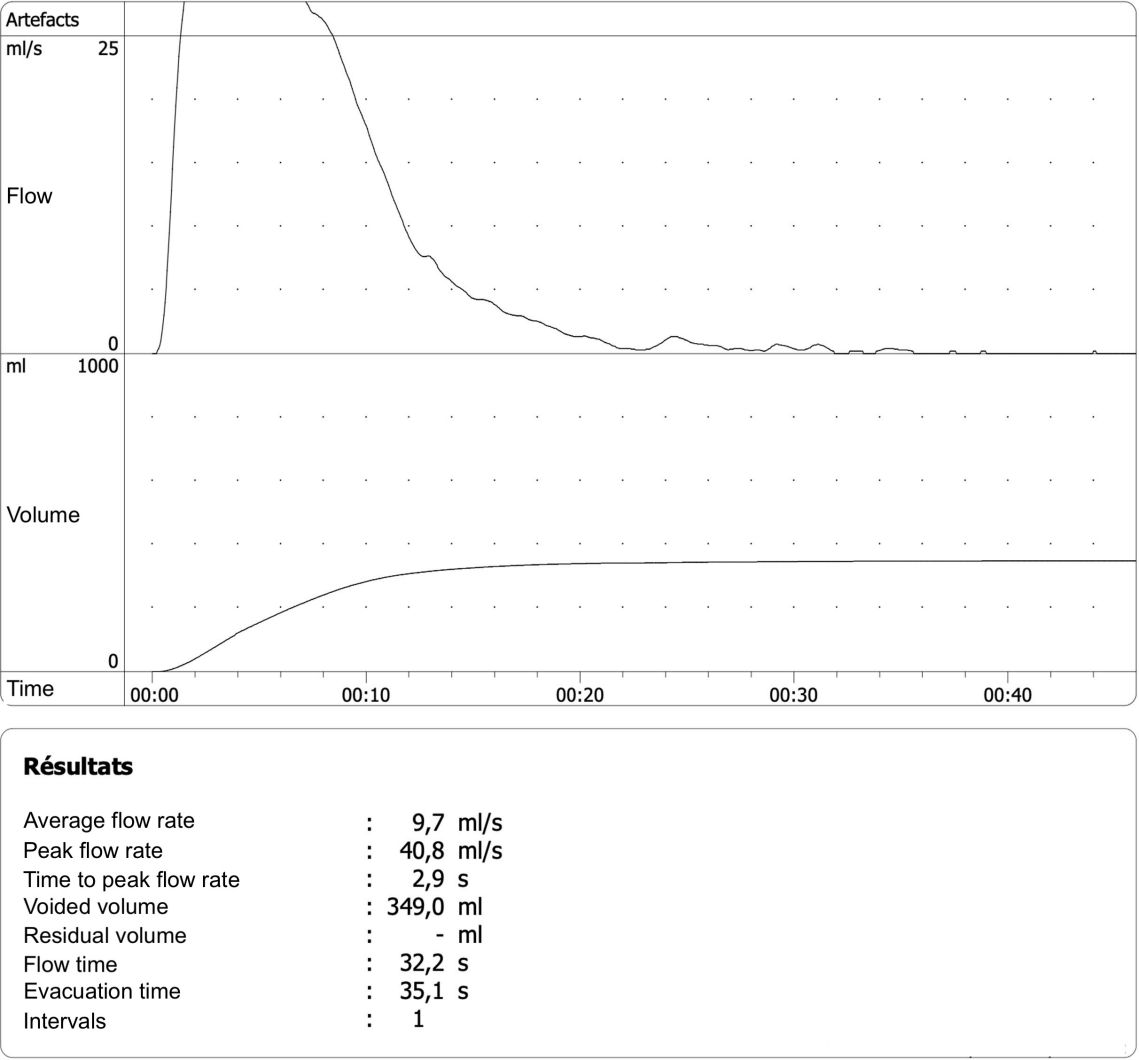
Debimetry 3-years after PAE showing excellent values with a peak velocity of 40.8 mL/s.

After 3 months, prostate volume was 142 ml (- 46%) and Qmax 34 ml/s. PSA lowered to 2.47 ng/ml. IPSS score was 1 and QoL score was 0. After 1 year, the prostate volume was 166 ml with a Qmax of 24.3 ml/s, IPPS score was 0, QoL was 0, there was no post-void residual (PVR) and PSA was 3.84 ng/ml. After 2 years, the prostate volume was 191 ml with a Qmax of 20.2 ml/s, IPSS score was 0, QoL was 0, there was no PVR and PSA was 3.84 ng/ml. After 3 years, the prostate volume was 199 ml with a Qmax of 40.8 ml/s (**Figure 4**), IPSS score was 0, QoL was 0, there was no PVR and PSA was 4.56 ng/ml. After 4.5 years, the prostate volume was 205 ml with a Qmax of 19.4 ml/s, IPSS score was 0, QoL was 0, there was no PVR and PSA was 5.31 ng/ml. The small increase of the prostate volume is probably due to regrowth, in keeping with the slight re-elevation of PSA. However, there was no need of reintroduction of medication or other measure during the whole follow-up. There was no change in the sexual function after PAE, especially no retrograde ejaculation.

Unfortunately, it has not been possible to organize another clinical visit, but the patient recently confirmed us during a telephonic consultation that he was still free of LUTS (IPSS 1, QoL: 0), more than 5 years after PAE.

## Discussion

GPH is defined as a hypertrophied prostate with a volume larger than 200 mL. Transurethral resection of the prostate (TURP) is classically reserved to prostate volume less than 80 ml (5). Patient suffering from GPH have limited options and the gold standard therapy, open adenomectomy, bears complications such as blood loss requiring transfusion, urinary incontinence, urinary bladder neck stenosis, and urethral stricture. Furthermore the total prostate volume seems to be associated with increased surgical difficulty (5, 6). PAE is an alternative to surgical treatment, especially for large prostate (>80 mL) (7). This technique has no upper size limit, in contrary to TURP and other minimally invasive options that are reserved for smaller adenomatous glands (8). It can be performed in an outpatient setting.

PAE should especially be regarded for patients with comorbidities and contraindication for general anesthesia because embolization is typically performed under conscious sedation and the bleeding risk is very low. It is also well suited for younger, sexually active patients who have concerns about retrograde ejaculation (a frequent consequence of TURP in over 75% of patients), erectile dysfunction or urinary incontinence (9).

Carnevale et al. published recently a large retrospective study on 316 men treated by PAE for LUTS due to BPH with a mean prostate volume of 93 ml (range: 30-330 ml) (10). After embolization (mean follow-up of 27 months), the mean reduction of prostate volume was 39%. The IPSS and QoL score improved by an average of 16 points and 4 points, respectively. No patient experienced urinary incontinence or erectile dysfunction (10). Those results are supported by a recent review and meta-analysis on 1254 patients suggested that PAE can reduce moderate to severe LUTS in men with BPH with a low risk of complications (11). From the patients’ perspective, prostatic artery embolization is a well-tolerated method for treating benign prostate hyperplasia (1). A recent retrospective study of 72 giant (> 200 mL) prostatic hyperplasia treated by PAE showed excellent outcome at 24 months with mean IPSS decreasing from 26.5 to 10.5 (p < 0.01), mean QoL decreasing from 6.0 to 2.0 (p < 0.01), mean Qmax increasing from 8.0 to 18 ml/s (p < 0.01) and mean PV decreasing from 303.0 ml to 209.0 ml (p < 0.01). No major complication was recorded. Thus, GPH appears as an excellent indication for PAE (4).

To our knowledge no comparative data exist between PAE and open adenectomy. Three randomized control trials comparing TURP and PAE showed the no inferiority and a better safety profile and lower cost of PAE (12–14). Moreover, PAE is associated with fewer adverse effects and shorter hospitalization times than transurethral resection (15).

Durability of the therapeutic effect is often considered as a major downside of PAE. In Carnevale et al. study, 63% of the men were free of LUTS recurrence at 60-month follow-up and LUTS recurrence was 23% at a mean follow-up of 27 months. However, none of the men with recurrent symptoms presented urinary retention and repeat of PAE was successfully done in 17% of the recurrence of LUTS with good clinical results (10). Even if data is lacking regarding long term recurrence after PAE, it doesn’t contraindicate a potential future surgical resection. Sare et al. even reported that PAE before surgery might reduce perioperative bleeding and operative time (16). It is also interesting to note that other embolic agent, especially cyanoacrylate, is currently evaluated for better long term effect (17).

Identifying the ‘ideal’ patient for PAE is a crucial area of research since 10 to 20% of patients do not respond to PAE despite technical success. Younger patients (below 70 years), those with acute urinary retention, IPSS lower than 25 points, central gland to total volume ratio greater than 50%, central gland adenomas of 1cm or greater, tend to do well after PAE. However, patients with pedunculated median lobes tend to not respond as well to PAE (11). Furthermore, patients with large middle lobes seem not to be ideal candidates as compared to those with enlargement primarily occurring in the lateral lobes probably because a ball valve component of obstruction. Thus, patient selection seems to be crucial for the technique (18). A prostate volume under 40 mL or advanced signs of obstructive urinary disease (e.g. hydronephrosis, large bladder diverticula, bladder stones) are relative contraindications and surgery might be a better option in these cases. Finally, severe pelvic atherosclerotic disease may imper catheterization.

Our case illustrates that PAE seems to be an efficient treatment for GPH with recurrent acute urine retention episode necessitating temporary indwelling catheter. Our patient had excellent improvement of symptoms (IPSS and QoL). The debimetry (Qmax) rose after PAE to reach a peak at 40.8 ml/s Prostate volume reached a nadir at 142 ml (- 46%). There was no recurrence of acute urine retention episode. We observed a moderate regrowth of the prostate, but interestingly there was no worsening of the symptoms or of the debimetry. In case of reappearance of LUTS, a new session of PAE or a more invasive urologic intervention would still be possible.

The growing body of literature assessing PAE is increasing and will help clarify the future role of PAE in the management of LUTS from prostatic hyperplasia. The European Association of Urology states that PAE is less effective than TURP and that it could be offered as an alternative to patient accepting less optimal outcomes (3). However, the trend is slowly changing and NICE has been recently supporting PAE in its guidelines (19). Thus, the radiologist has a crucial role for working along with urologist to select optimal patient and follow them after PAE. Among these, patients with GPH may be excellent candidates for PEA (19).

## Conclusions

We presented a case of PAE for a GPH (265 ml) in a young (54-years old) patient with LUTS and history of several episode of acute urinary retention. Clinical outcome was excellent and there is still no symptom of LUTS more than 5 years after the intervention. Further research and long-term follow-up is needed in order to further define the gold standard treatment for GPH and symptomatic LUTS.

## Data Availability

The original contributions presented in the study are included in the article/supplementary material. Further inquiries can be directed to the corresponding author.
